# Enhancing insecticidal efficacy of *Bacillus thuringiensis* Cry1Ab through pH-sensitive encapsulation

**DOI:** 10.1007/s00253-023-12723-w

**Published:** 2023-08-26

**Authors:** Elham Jalali, Yolanda Bel, Shahab Maghsoudi, Ebrahim Noroozian, Baltasar Escriche

**Affiliations:** 1 Department of Chemistry, Shahid Bahonar University of Kerman, Kerman, 76169-14111 Iran; 2 Instituto BioTecMed, Department of Genetics, University of Valencia, 46100 Burjassot, Valencia Spain

**Keywords:** Nanomaterials, European corn borer, Bioassay, Bioprotectant, Cry toxicity, Environmental degradation

## Abstract

**Abstract:**

Nanotechnology is a promising way to enhance the stability of *Bacillus thuringiensis* (Bt) insecticidal proteins under environmental conditions. In this work, two emulsions were prepared through the Pickering emulsion technique, stabilized by Cu^2+^-SQDs/S-CN nanocomposites and by GO nanosheets. In addition, a pH-sensitive polymer was incorporated into these emulsions, allowing the Bt protein, Cry1Ab, to be released in an alkaline pH environment, as it occurs in the lepidopteran pests’ gut. The effectiveness of these two nanomaterials in protecting Cry1Ab from degradation, and therefore enhancing its pesticidal activity, was assessed by exposing samples of the purified unprotected protein and encapsulated protein to high-intensity UV light and 40°C temperature treatments. The UV treatment results were evaluated using SDS-PAGE analysis and pointed out that Cry1Ab could be structurally protected by the emulsions. The bioassays with first instar larvae of the lepidopteran pest *Ostrinia nubilalis* confirm the nanomaterial protection to UV and temperature treatments, i.e., decreasing about half the degradation rate and increasing up to 12-fold the residual activity after UV treatment. Our results indicate that encapsulation could be an effective strategy to improve the effectiveness of Cry1Ab under environmental conditions.

**Key points:**

• *Pickering emulsions are effective for solubilized Cry1Ab encapsulation.*

• *Structural and toxicity Cry1Ab properties are enhanced by pH-sensitive encapsulation.*

• *Cu*^*2+−*^*SQDs/S-CN and GO nanomaterials improve the efficacy of Bt insecticides.*

**Supplementary Information:**

The online version contains supplementary material available at 10.1007/s00253-023-12723-w.

## Introduction

In recent decades, many efforts have been done aimed at developing sustainable plant protection products, especially microorganism-based biopesticides. *Bacillus thuringiensis* (Bt)-based pesticidal products have been used worldwide successfully for decades (Damalas and Koutroubas 2018; Samada and Tambunan 2020). *B. thuringiensis* (Bt) is a Gram-positive spore-forming bacterium that produces during sporulation insecticidal proteins (Cry and Cyt), which are accumulated in crystal inclusions (Palma et al. 2014). The Cry proteins are produced by Bt, as protoxins. After being ingested by the target insect, the protoxins are solubilized within the insect gut, due to the highly alkaline pH, and are activated by midgut proteases to release the toxin fragment that eventually causes insect death (Parker and Feil 2005). The major drawback of using Bt proteins as active ingredients in biopesticides is the lack of persistence in nature due to environmental conditions, such as ultraviolet (UV) radiation, high temperatures, and rain, all of which can cause a reduction in the insecticidal activity or can inactivate Cry activity (Leong et al. 1980; Peralta and Palma 2017; Fernández-Chapa et al. 2019). Indeed, UV light can give rise to the inactivation of crystal proteins by destroying their three-dimensional (3D) structure (Zhang et al. 2021) and therefore disrupting their solubilization in the alkaline environment of the insect midgut leading to severe repercussions for product stability (Cui et al. 1993). Also, it is possible that the temperature indirectly accelerated or inhibited the degradation of crystal proteins by indirectly controlling soil microbe activity (Yao-yu et al. 2007).

Nanomaterials can be used in protecting Bt because of their unique properties such as their small size, excellent dispersion, and their large surface area (Vidallon and Teo 2020). Several studies have focused on inorganic metal oxide nanoparticles’ application as a UV absorber in pesticides, and their results have demonstrated that the use of nanoparticles can improve pesticide properties (Erdem et al. 2010; Prasad et al. 2014; Kumar et al. 2019). By using Fe_3_O_4_ nanoparticles in colloidosomal microcapsule formulations, the bioactivity of Bt was successfully extended for the management of lepidopteran pests of *Brassica* crops when compared to the commercial formulation (Bashir et al. 2016). In previous works, Pickering emulsions combined with nanomaterials have been studied and applied as a promising and effective strategy for extending eco-friendly Bt insecticide (Bashir et al. 2016; Jalali et al. 2020; Koroleva and Yurtov 2022). Jalali et al. (2020) showed the successful protective effect of the GO nanosheets on the stability of Bt formulations exposed to UVA radiation. Regarding individual Cry proteins (not included in Bt crystals or formulations), their protection is a fundamental strategy since these toxins play the leading role in the target organism’s mortality (Swamy and Asokan 2013; Vimala Devi et al. 2019). Loading of Cry11Aa toxins on Mg(OH)_2_ nanoparticles indicated that nanoparticles, as an excellent nanocarrier, could substantially improve the anti-ultraviolet resistance and insecticidal bioactivity of the Cry11Aa protein (Pan et al. 2017).

As an effective strategy for designing biopesticides, emulsions have been employed to encapsulate, protect, and enhance Bt insecticidal properties. Encapsulation can take place with a variety of synthetic and natural polymers, as well as carbohydrates, waxes, gums, and fats. There are some downsides to the methods used to fabricate biopesticide microcapsules, such as the need for potentially toxic solvents, surfactants, and/or high temperatures. Pickering emulsions have recently emerged as an excellent method for stabilizing emulsions. They are stabilized by adsorbing nanoparticles at the oil–water interface to prevent the coalescence of the emulsion droplets. Pickering emulsion is a promising way to trap a compound in a material stabilized by interfacial nanoparticles to reduce the damaging effect of environmental conditions. As compared to conventional emulsions stabilized by surfactants, Pickering emulsions are more stable, less toxic, and exhibit greater interfacial adsorption energy, allowing for irreversible adsorption. There are nanomaterials with unique properties that can be used for stabilizing emulsions. Graphene oxide (GO) is an oxidized form of graphene that received special attention due to its high surface-area-to-volume ratio, low cost, exceptional thermal, electrical, mechanical, and optical properties, and unique atomic structure. These properties of GO nanosheets make them promising nanomaterials for bio-applications (Yang et al. 2021).

Graphitic carbon nitride (g-CN) has attracted more attention for several applications because of its chemical and thermal stability, excellent biocompatibility, high activity, high fluorescence, and efficient visible-light absorption (Zhang et al. 2016). Doping of g-CN with some elements, such as sulfur, has been regarded as an efficient technology for increasing the optical sensitivity of g-CN in the visible spectral region. The homogeneous substitution of lattice nitrogen by sulfur and concomitant quantum confinement effects make the electronic structure in S-CN unique (Kadam et al. 2019). Sulfur quantum dots (SQDs) with their significant biological activities and unique properties can be prepared as a new class of photoluminescent nanomaterial from the inexpensive precursor (Shen et al. 2018). This study aimed to report a new and straightforward method for the development and synthesis of highly fluorescent SQDs by treating sublimated sulfur powder and NaOH using polyethylene glycol-400 (PEG-400) as a ligand. Since the SQDs synthesized using this method have poor photostability, Cu^2+^ was applied to modify the highly luminescent SQDs. In this regard, Cu^2+^-SQDs were prepared via a surface modification method, and Cu^2+^-SQDs /sulfur-doped graphitic carbon nitride (Cu^2+^-SQDs/S-CN) nanocomposites were synthesized via a one-step hydrothermal strategy.

This paper aims to provide a novel method for the encapsulation of purified and solubilized Cry1Ab protoxin (more unstable to environmental changes than Cry1Ab protein in the parasporal crystals) via the Pickering emulsion stabilized by two different nanomaterials: GO nanosheet and the new Cu^2+^-SQDs/S-CN nanocomposites. These microparticles were designed with a pH-sensitive polymer so that they could release Cry1Ab in the alkaline environment of the larval midgut. The performance of these two nanomaterials in protecting the integrity of the Cry1Ab after ultraviolet (UV) radiation and high temperatures was investigated, checking its toxicity against first instar larvae of the susceptible lepidopteran pest *Ostrinia nubilalis*.

## Material and methods

### Preparation of sulfur-doped graphitic carbon nitride nanosheets

The bulk sulfur-doped graphitic carbon nitride nanosheets (S-CN) were synthesized via a simple calcination method following Wang et al. (2015). Briefly, 10 g thiourea was put into a covered crucible in a muffle furnace and calcined at 550 ℃ for 4 h at a ramping rate of 5 °C min^**−**1^. The resulting light-yellow powder was washed and dried at 50 °C in a vacuum oven. After that step, 1.2 g of bulk S-CN was milled into powder in an agate mortar and then added to 40 mL of methanol. After ultrasonic treatment for 2 h, the bulk S-CN was exfoliated into thin nanosheets, which were washed several times with deionized water and then dried.

### Synthesis of the sulfur quantum dots etched using Cu^2+^

The synthesis of the sulfur quantum dots etched using Cu^2+^ (Cu^2+^_-_SQDs) was performed according to the synthesis method reported by Shen et al. (Shen et al. 2018). The sublimated sulfur powder (1.4 g), PEG-400 (3 mL), sodium hydroxide (4.0 g), and 50 mL of ultrapure water were mixed and subject to reflux at a constant temperature reaction at 70 °C under continuous stirring for 72 h. The sublimated sulfur powder was gradually dissolved, and over time its color change to dark red was observed. The resulting product was centrifuged at 1538 × *g* and referred to as SQDs. The SQDs (1.5 mL) and 0.6 mmol of Cu^2+^ were introduced into a round bottom flask and mixed at 70 °C for 2 h under vigorous stirring. The product had a deep yellow color and after filtration, the mixture became light yellow. The resulting products were referred to as Cu^2+^-SQDs.

### Synthesis of Cu^2+^-SQDs/S-CN nanocomposite

The S-CNs and SQDs previously prepared were used to synthesize the nanosheets (Cu^2+^-SQDs/S-CN) (Wang et al. 2015; Zhang et al. 2021). In summary, 0.1 g of S-CN powder and 1 mL of Cu^2+^-SQDs solution were added to 25 mL ultrapure water, and the mixture was further stirred for 30 min at room temperature. Subsequently, the mixture was sealed into a Teflon-lined autoclave and maintained for 4 h at 140 °C. Finally, the Cu^2+^-SQDs/ S-CN was collected, washed with deionized water, and then dried at 60 °C for 12 h.

### Synthesis of GO nanosheets

The GO nanosheets were synthesized from natural graphite powder by a modified Hummer’s method as reported previously (Maghsoudi and Jalali 2017). In short, the graphite powder (2.5 g) and NaNO_3_ (1.25 g) were added to H_2_SO_4_ (57.5 mL) in an ice bath under vigorous stirring. Next, under stirring, KMnO_4_ (7.5 g) was slowly added to the suspension. The system was kept under stirring at room temperature and diluted in water (115 mL), followed by DI water (350 mL) and H_2_O_2_ (15 mL) addition. Then, the resulting suspensions were washed several times with HCl (5%), and DI water to reach pH = 5. Finally, the suspension was laminated into GO nanosheets by using a titanium-alloy solid ultrasonicator (20 kHz, 400 W, UP400st, Hielscher, Germany), and freeze-dried for 48 h.

### Cry1Ab protein preparation

The Cry1Ab protein was obtained from a recombinant *Escherichia coli* strain kindly provided by Dr. R. de Maagd (Wageningen University, the Netherlands). The Cry1Ab protoxin expression in *E. coli* strain XL-1 Blue, solubilization, and lyophilization were performed following Herrero et al. (2004). In brief, *E. coli* was grown in a Terrific Broth (TB) medium supplemented with 50 µg/mL of ampicillin and 2% glucose, at 37°C. The cell culture was centrifuged at 12,000 × *g* for 8 min at, 4 °C, and the pellet was resuspended in lysis buffer (3 mL of 50 mM Tris–HCl [pH 8.0], 5 mM EDTA, 100 mM NaCl per gram of pellet). Then, 8 µL of 50 mM phenylmethylsulfonyl fluoride (PMSF) and 800 µg of lysozyme were added per gram of pellet. After incubation at room temperature for 20 min, 1 mg/mL of deoxycholic acid was added, and the culture was incubated at 37°C for 30 min. After that step, 50 µg of DNase I/mL was added followed by 30-min incubation at 37°C. The mixture was sonicated for 20 s at half max output (Bandelin SonoPlus-HD2070, Germany) and centrifugated at 39,000 × *g* for 20 min. The pellets containing the protoxin inclusion bodies were washed several times with 20 mM Tris–HCl [pH 7.5], 1% Triton X-100, and 1 M NaCl. For solubilization of the inclusion bodies, the pellet was incubated at 37°C in the solubilization buffer (50 mM sodium carbonate, pH 10.0) containing 10 mM dithiothreitol. After 2h, the sample was centrifuged at 39,000 × *g* for 20 min and the soluble protoxin in the supernatant was stored at 4°C. For long-term storage, the Cry1Ab protoxin was lyophilized using a Thermo Savant Modulyo D-230 Lyophilizer. Before each use, the solubilized Cry1Ab was quantified by the method of Bradford (Bradford 1976) using BSA as standard, and its integrity was analyzed by sodium dodecyl sulfate–polyacrylamide gel electrophoresis (SDS-PAGE).

When necessary, protein concentrations were evaluated by densitometry after visualization by SDS-PAGE, using bovine serum albumin (BSA) as standard. The densitometry analyses were performed using the TotalLab Quant version 12.3 program (Newcastle, UK).

### Synthesis of poly (methyl methacrylate-co-methacrylic acid) (P(MMA-co-MA))

The polymer was prepared as described previously (Jalali et al. 2020). Briefly, methyl methacrylate (10.65 g), methacrylic acid (4.24 g), ammonium persulfate (0.12 g), sodium dodecyl sulfate (0.20 g), and 50 mL deionized water (DI) were mixed and stirred at 350 rpm and degassed by bubbling nitrogen for 30 min. Then, the mixture was heated to 80 °C under a nitrogen atmosphere. After 10 h, the P(MMA-co-MA) particles were collected. The P(MMA-co-MA) particles can be dissolved in an alkaline environment easily (Jalali et al. 2020).

### Preparation of Pickering emulsion stabilized by nanomaterial

Dispersions of nanomaterials (0.15% w/v) in olive oil and ethanol were prepared by using an oil phase containing nanomaterial dispersion and applied via a homogenizer with a 3-mm dispersion probe for 3 min. The aqueous phase solution was prepared by dissolving Cry 1Ab protein (1.6% w/v) in 250 µL p(MMA-*co*-MA) and then adding 250 µL DI water and 0.0073g NaCl. The mixture was homogenized for 1 min. The Pickering emulsion was prepared by adding the oil phase dropwise to the aqueous phase and ultrasonicated at room temperature to obtain a uniform emulsion for 2 min. The volume ratios of the oil phase to the aqueous phase tested were 1:1, 2:1, 3:1, 4:1, 5:1, and 10:1.

### Insect rearing and bioassays

The European Corn borer, *O. nubilalis* (Lep.: Crambidae), was used in this study to assess the toxicity of the Cry1Ab protein. The insect colony had been maintained for more than 4 years at the insect-rearing facilities of the Department of Genetics of the University of Valencia (Spain). The larvae were reared on an artificial diet (Poitout and Bues 1974) under 25 ± 1 °C, 70 ± 5% relative humidity, and with 16:8 h L:D photoperiod controlled conditions, and the adults were fed with honey diluted in water.

Bioassays to assess the dose–response were conducted using the diet surface contamination method (Beegle 1990). Seven concentrations of protein were tested in each bioassay performed. The protein for the bioassays consisted of serial dilutions of non-encapsulated Cry1Ab or serial dilutions of encapsulated Cry1Ab, previously quantified by Bradford. The protein solubilization buffer (50 mM sodium carbonate, pH 10.0) was used as the negative control. Bioassays to determine mortality parameters were completed scoring the mortality after 7 days. For each concentration of each replicate, 16 neonate larvae were used. Three biological replicates were performed for each experiment.

### UV treatments

The UV light treatments consisted in subjecting the Cry1Ab samples to UVC irradiation, from 6 × 8 W UV tubes emitting at a primary wavelength of 254 nm, in a CL-1 UV model cross-linker (Herolab, Germany). The samples were located at a distance of 12 cm from the UV source and exposed at different times.

UVC radiation has more damaging effects than the predominant UVA and UVB ones. We choose that radiation to simulate a harsher environment than in an open field. The effects of UVC on the encapsulated and non-encapsulated Cry1Ab protein were tested in two ways: (1) By SDS-PAGE after measuring the protein concentration by densitometry; (2) by single-dose bioassays using a fixed concentration of 3.3 ng/cm^2^ (LC_50_ value) of Cry1Ab in the samples.

### Temperature treatments

Cry1Ab samples were incubated at room temperature (about 25°C, RT) and 40°C (HT) in an incubator (Termo shaker TS-100 Biosan, Riga, Latvia). The samples were treated for 15, 30, 60, 120, 300, 960, and 1440 min. Single-dose bioassays using a fixed concentration of 3.3 ng/cm^2^ (LC_50_ value) of Cry1Ab were performed to assess the protein toxicity after the temperature treatments.

### Statistical analyses

Mortality values obtained from the time-course exposition experiments (Figs. 5 and 6) were normalized regarding the mortality obtained at 0 min for better visual comparison. Statistical analyses were performed based on the number of insects assayed. Bioassays using a range of Cry1Ab concentrations were analyzed using the POLO-PC software program (LeOra Software, Berkeley, CA, USA, 1987) which performs a Probit analysis (Finney 1971) which estimates the parameters of the dose–response quickness (slope) and the concentration that killed 50% of treated insects (LC_50_), with their respective standard error of the mean (SE) and the fiducial limits at 95% (FL_95_). LC_50_ values were considered significantly different if fiducial limits did not overlap.

The degradation of a pesticide is proportional to the concentration of the product and can be inferred from the toxicity (mortality) data using a first-order reaction as it is suggested by different authors and the United States Environmental Protection Agency (US EPA n.d) (Walker and Barnes 1981; Wu and Nofziger 1999). The equation describes the process of reducing an effective toxin amount by a consistent percentage rate over a period of time. The degradation rate constant (half-life) would not change with time under environmentally fixed conditions. The equation is formulated as *Y* = (S*e^*KX*^) + *R*, where *X* is time and *Y* is the response (mortality). The parameter *R* estimates the residual activity and the *S* (span) one corresponds to the difference between *R* and the *Y* initial value (*Yo*). The *Yo* value decreases to *R* with a rate constant (*K*), from which calculates the half-life of the product (time in which half of the initial product has degraded) as *e*^2^/*K*. Differences in dead larvae after protein treatments and parameter values were tested by a one-way ANOVA, followed by a Tukey’s post-test, considering *P* < 0.05 as a significant difference. GraphPad Prism 7.0 (GraphPad Software, La Jolla, CA, USA) was used to perform all these analyses.

## Results

### Structural characterization of nanoparticles

**Fig. 1:**
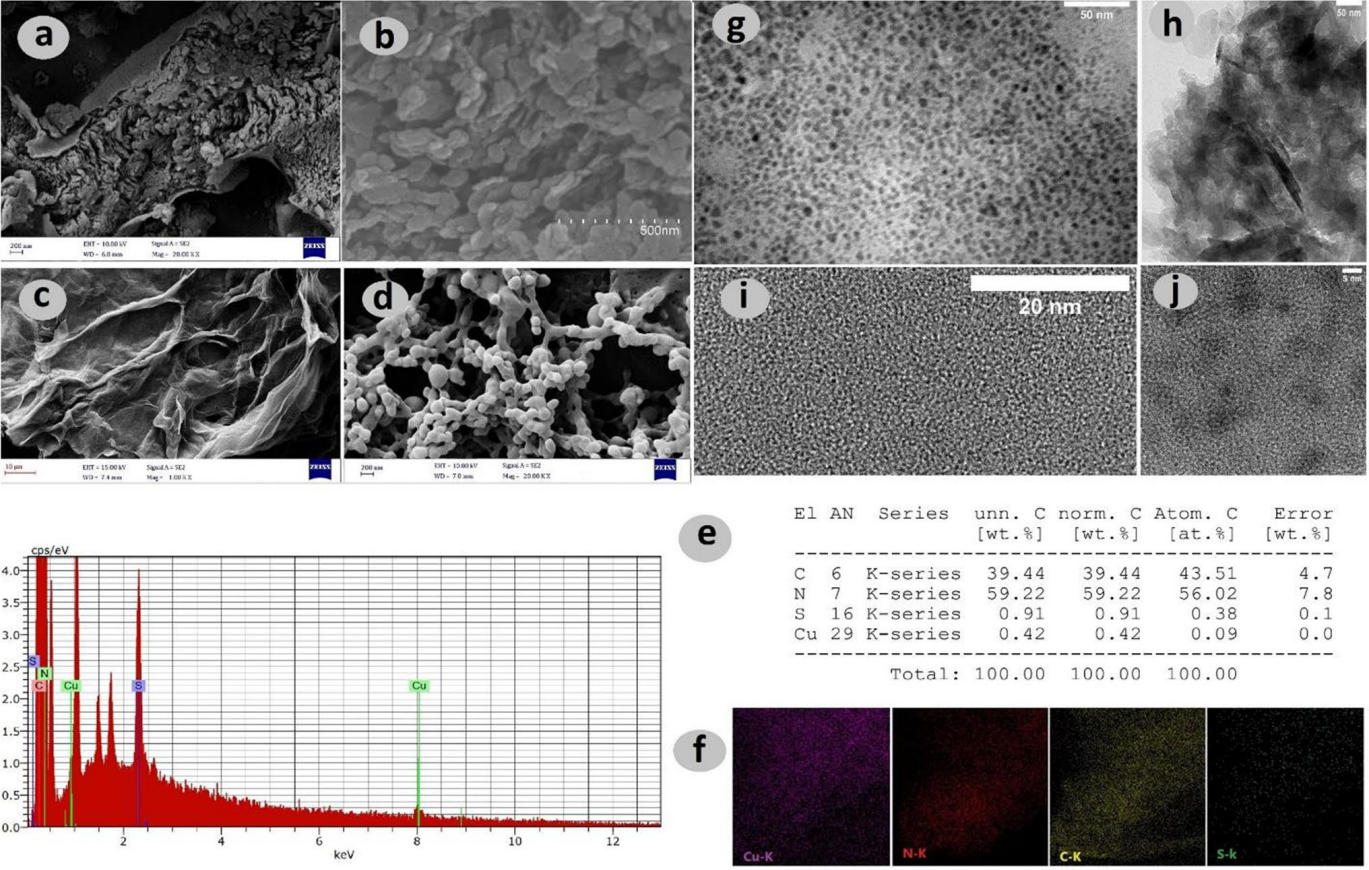
FE-SEM image of (**a**) S-CN nanosheets (scale bar: 200 nm), (**b**) Cu^2+^-SQDs/S-CN nanocomposite (scale bar: 500 nm), (**c**) GO nanosheets (scale bar: 10 µm), (**d**) P(MMA-*co*-MA) (scale bar: 200 nm), (**e**) EDX spectrum of Cu^2+^-SQDs/S-CN nanocomposite, (**f**) elemental mapping images of Cu^2+^-SQDs/S-CN nanocomposite, (**g**) TEM images of Cu^2+^-SQDs (scale bar: 50 nm), (**h**) Cu^2+^-SQDs/S-CN nanocomposite (scale bar: 50 nm), (**i**) HRTEM images of Cu^2+^-SQDs (scale bar: 20 nm), and (j) Cu^2+^-SQDs/S-CN nanocomposite (scale bar: 5 nm)

The surface morphology and microscopic structures of the S-CN nanosheets, Cu^2+^-SQDs/S-CN nanocomposite, GO nanosheets, and p(MMA-co-MA) were investigated using field emission scanning electron microscopy (FE-SEM), and the results are summarized in Fig. 1.

The FE-SEM image (Fig. 1a) revealed the hierarchical morphology comprising regular crumpled nanosheets of S-CN. Cu^2+^SQDs/S-CN nanocomposite was made from many nanosheets (Fig. 1b). Also, due to the small particle size of the Cu^2+^-SQDs, it was not possible to observe Cu^2+^-SQD in Cu^2+^-SQDs/S-CN nanocomposite by FE-SEM. The results suggested that after the decoration of Cu^2+^-SQDs, the basic structure of S-CN had not changed. On the other hand, the layers’ structure of the 2D GO sheets with numerous wrinkles and folds on the surface (Fig. 1c) and FE-SEM image (Fig. 1d) of the hollow structure of p(MMA-co-MA) showed that the aggregation of p(MMA-co-MA) caused the stabilization of the emulsion.

To investigate the elemental composition and to determine the percentage of each element in the Cu^2+^-SQDs/S-CN nanocomposite, energy dispersive X-ray (EDX) spectroscopy was used (Fig. 1e) and the elemental mapping images of Cu^2+^-SQDs/S-CN nanocomposite are shown in Fig. 1f. Both analyses confirmed the presence of C, N, S, and Cu elements in the nanocomposite, indicating the successful decoration of the S-CN by Cu^2+^-SQDs.

The successful synthesis of Cu^2+^-SQDs with quantum-sized sphere morphology was confirmed by transmission electron microscopy (TEM) images (Fig. 1g) and high-resolution transmission electron microscopy (HRTEM) (Fig. 1i). The TEM of Cu^2+^-SQDs/S-CN nanocomposites (Fig. 1h) indicated that the SQDs were homogeneously dispersed across the S-CN nanosheet surface. The successful Cu^2+^-SQD decoration onto S-CN without any change in the basic structure of QDs was revealed by the HRTEM image (Fig. 1j) with black dots with diameters ranging from 2 to 10 nm. Therefore, it could be confirmed that the Cu^2+^-SQDs/S-CN nanocomposite was successfully synthesized through hydrothermal treatment.

**Fig. 2:**
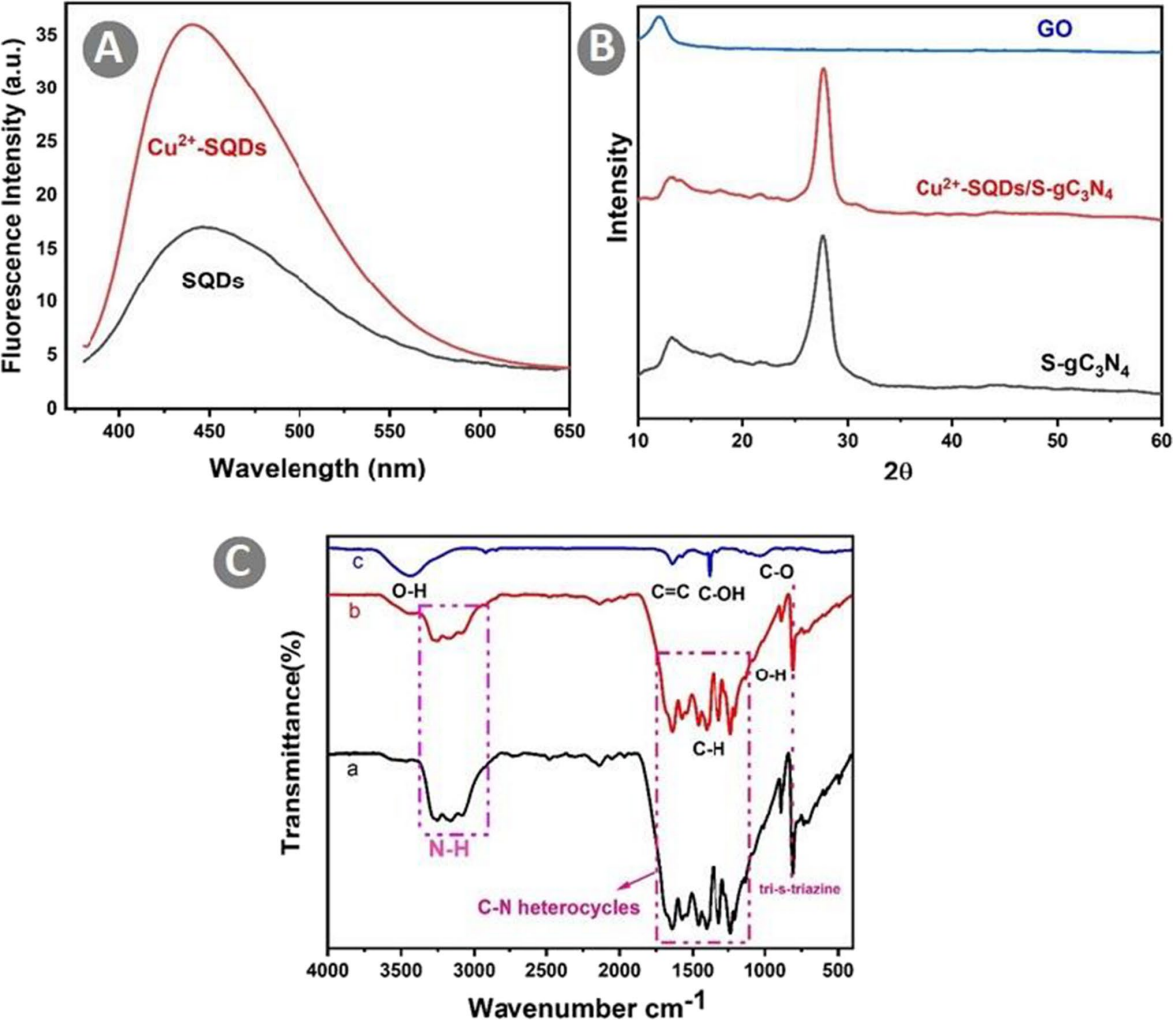
Fluorescence spectra of (**A**) the S dot particle before etching and Cu^2+^-SQDs; (**B**) XRD pattern of Cu^2+^-SQDs, Cu^2+^-SQDs/S-CN nanocomposite, and GO nanosheets; (**C**) FT-IR spectra of (a) Cu^2+^-SQDs, (b) Cu^2+^-SQDs/S-CN nanocomposite, and (c) GO nanosheets

The effect of Cu^2+^ etching on SQDs could be observed after increasing its fluorescence intensity under the radiation of a UV lamp at 365 nm and emitting a remarkable blue fluorescence (Fig. 2A). This confirmed that the Cu^2+^ application could enhance the SQD fluorescence. As the fluorescence increases, the possibility of UV light absorption will increase more effectively, resulting in less damage to the Cry1Ab protein.

X-ray diffraction (XRD) analysis was performed to investigate the phase structures of the samples (Fig. 2B). Two diffraction peaks at 13.1° and 27.7° corresponding to the (100) and (002) crystal planes were observed for S-CN and Cu^2+^-SQDs/S-CN. The peak at 27.6° was related to the stacking structure of conjugated aromatic rings. The peak at 13.1° resulted from the in-plane repeating unit of tri-s-triazine. The XRD patterns of Cu^2+^-SQDs/S-CN and S-CN samples were highly similar (Fig. 2B), pointing to no characteristic peaks of Cu^2+^-SQDs, probably due to the low mass loading of Cu^2+^-SQDs on the nanocomposite. Finally, the XRD analysis of the GO nanosheets showed a sharp and strong diffraction peak at 12.04°, corresponding to the (001) plane of interlayer distance 0.73 nm. Successful oxidation of graphite into GO nanosheets was confirmed by the high intensity of this peak.

The Fourier transform infrared (FT-IR) spectra of S-CN, Cu^2+^-SQDs/S-CN, and GO nanosheets (Fig. 2C) showed that S-CN displayed some peaks in 1200–1650 cm^−1^ (Fig. 2C, line a), a range attributed to the C-N stretching modes of the carbon nitride heterocycles. A broad absorption band was located in the range of 3000 to 3500 cm^−1^, corresponding to the NH vibrational stretching modes. The peak at 807 cm^−1^ arose from the out-of-plane bending vibration of tri-s-triazine rings, indicating that there were tri-s-triazine units in the structure of the S-CN. The FT-IR spectrum of the Cu^2+^-SQDs/ S-CN (Fig. 2C, line b) showed the bands centered at 1460, and 942 cm^−1^ corresponding to the C–H bending and the stretching vibration of O–H of the PEG, respectively. This result indicated the presence of PEG on the surface of SQDs without any chemical reactions. Therefore, PEG was utilized as a passivating agent for the SQDs, restricting the aggregation of the SQDs and forming these with superior dispersibility. Because the amount of sulfur was too low, no peak was ascribed to the bond of sulfur with other elements. Besides, it was not observed significant peaks compared to pure S-CN when SQDs were incorporated (Fig. 2B and C). Hence, it could be confirmed that the main chemical structure of the nanocomposite was not altered when S-CN was coupled with the Cu^2+^-SQDs. According to Fig. 2B and C, compared to pure S-CN, no significant peaks could be observed when SQDs were incorporated. Hence, it could be confirmed that the main chemical structure of the nanocomposite was not altered when S-CN was coupled with the Cu^2+^-SQDs.

In the FTIR spectra of GO nanosheets (Fig. 2C, line c), the peaks in the range of 1620 to 1645 cm^−1^ were assigned to the C = C bond. The bands at 1030 cm^−1^ and 1383 cm^−1^ corresponded to C–O stretching vibrations of COOH groups and C–OH, respectively. The broadband at 3446 cm^−1^ was assigned to O–H stretching vibrations of the adsorbed water molecules of GO nanosheets.

### Encapsulation of Cry1Ab and toxicity against O. nubilalis

The Pickering emulsions were designed to encapsulate the purified and solubilized Cry1Ab protein. GO nanosheet and Cu^2+^-SQDs/S-CN nanocomposites were used to stabilize Pickering emulsion with good coalescence stability. The Cry1Ab protein would be protected from UV-induced photosensitive damage and temperature by these nanomaterials because of their properties of absorbing UV and temperature resistance. The best dispersion with the highest emulsion stability was achieved when the ratio of the oil to water phase is 3:1 (Figs. S1 and S2). It is worth noticing that as the oil/aqueous volume ratio increased from 1:1 to 3:1, the volume-averaged emulsion droplet size decreased. At a ratio of 4:1 of the oil/water phase, the droplet collapsed and this phenomenon was more prominent when the ratio increased to 10:1 (Figs. S1 and S2). In all the tested formulations, the formation of the capsule was confirmed upon microscopic observation. The emulsions were prepared with the fixed 3:1 ratio of oil/aqueous phase in subsequent experiments.

**Fig. 3:**
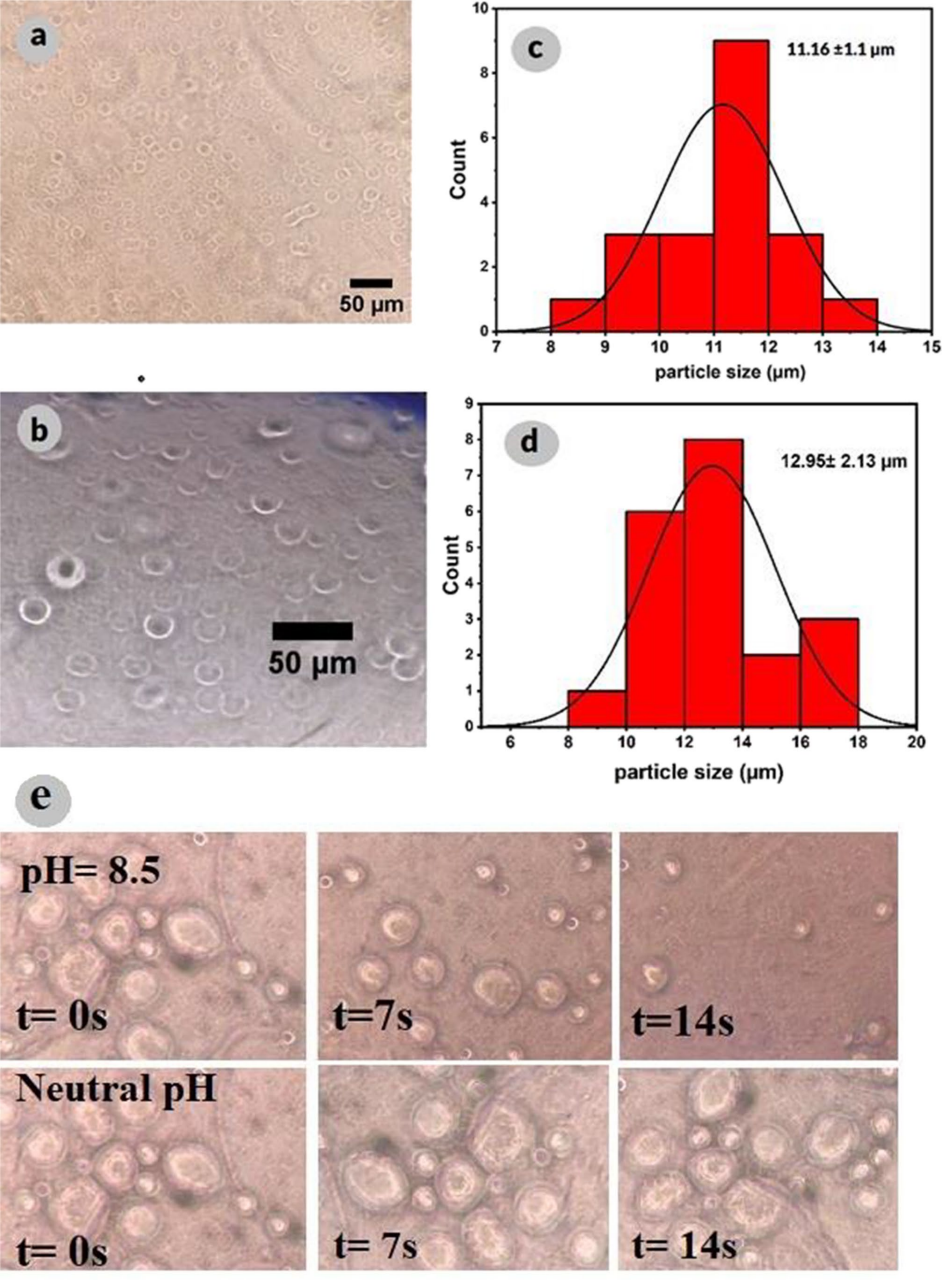
Optical microscopy pictures of (**a**) GO and (**b**) Cu^2+^-SQDs/S-CN particles and their histograms of capsule size distributions stabilized emulsion (**c**) GO and (**d**) Cu.^2+^-SQDs/S-CN, (**e**) dissolution of a capsule at pH = 8.5, and neutral pH observed by optical microscopy with a scale consistent with pictures a and b

The optical microscopy images (Fig. 3a and b) corroborate the spherical shape and polydisperse size distribution of both emulsions. The majority of capsules for GO and Cu^2+^-SQDs/S-CN stabilized emulsions ranged between 8.5 and 12 µm with an average of 11 ± 1.1 µm and 9.5 to 17 µm with an average of 13 µm ± 2 µm, respectively (Fig. 3c and d).

In addition, the release of capsule contents at pH 8.5 was investigated to provide pH conditions similar to those found in the larval midgut. As shown in Fig. 3e, the dissolution of the capsule wall at pH = 8.5 and neutral pH was observed by optical microscopy in about 14 s. A higher rate of release in alkaline than in neutral environments was evident from the microscopy images.

Bioassays with *O. nubilalis* first instar larvae were conducted to assess the toxicity of Cry1Ab. The toxicity results of the Cry1Ab in both free and encapsulated forms are summarized in Table 1. The Cry1Ab protein was found to be toxic for the *O. nubilalis*, with an LC_50_ value of 3.6 ng/cm^2^, in agreement with other published protoxin toxicity values (Farinós et al. 2004; Siqueira et al. 2004; Li et al. 2005; Crava et al. 2009).

**Table 1:** Toxicity parameters of Cry1Ab against *Ostrinia nubilalis* using encapsulated and non-encapsulated products

Cry1Ab product	LC_50_ (ng/cm^2^)	Fiducial limits (95%)	Slope ± SE
Non-encapsulated	3.59	2.40–5.18	1.01 ± 0.10
Cu^2+^-SQDs/S-CN emulsion	1.54	1.03–2.26	1.52 ± 0.14
GO emulsion	1.72	1.31–2.24	1.64 ± 0.15

After nanoencapsulation of Cry1Ab with Cu^2+^-SQDs/S-CN nanocomposite or with GO nanosheets, the LC_50_ values decreased slightly to 1.5 and 1.7 ng/cm^2^, respectively, indicating an increase in the insecticidal activity. The bioassays performed with nanomaterials (with any of them) without Cry1Ab did not cause mortality (the mortality values obtained were similar to the ones obtained in the negative controls, performed with just carbonate buffer), indicating that Cry1Ab was the only lethal agent. Therefore, the increase in toxicity observed in the encapsulated Cry1Ab samples was probably due to the combination of Cry1Ab with the encapsulation nanomaterials.

### UV protection

**Fig. 4:**
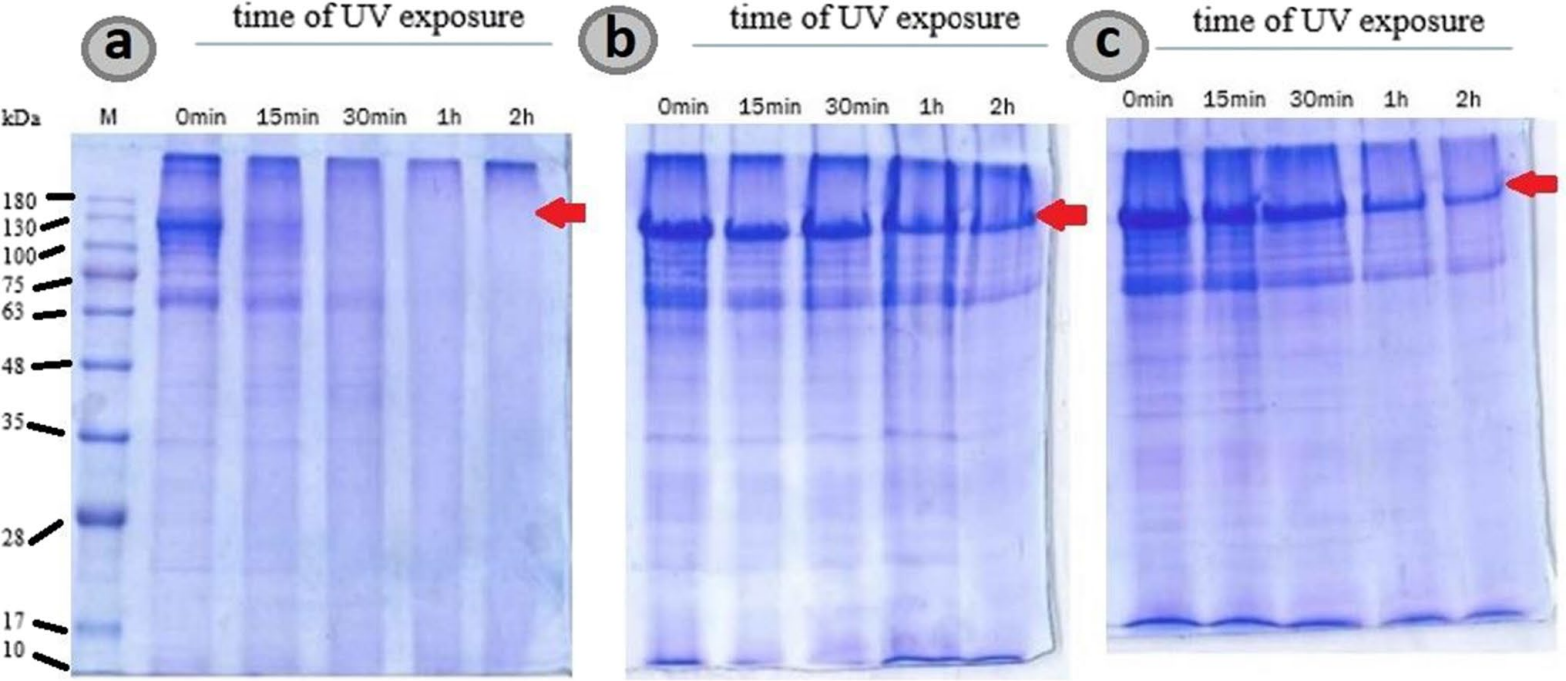
SDS-PAGE protein analysis from (**a**) non-capsulated Cry1Ab, (**b**) GO emulsion, and (**c**) Cu^2+^-SQDs/S-CN emulsion after UV radiation. The red arrow points to the Cry1Ab protein band

For evaluation of the protection of the Cry1Ab protein in emulsions stabilized with GO and Cu^2+^-SQDs/S-CN nanomaterials after UV exposure, SDS-PAGE analyses were performed, to check the Cry1Ab content in the samples after different treatment times (Fig. 4).

The UV light used was UVC, generally used for sterilization purposes, to subject the sample to the worst-case scenario. In the SDS-PAGE analyses, the Cry1Ab protein before treatments appeared as a band of about 130 kDa (protoxin form) together with a minor band of about 66 kDa corresponding to the activated protein (toxin) (Fig. 4a). After 15-min exposure to UVC, the Cry1Ab protoxin band was not observed in non-capsulated Cry1Ab protoxin (Fig. 4a), while, in both Pickering emulsions, Cry1Ab was stabilized by nanomaterials, and the bands corresponding to Cry1Ab proteins could be still detected after 2 h (Fig. 4b and c).

**Fig. 5:**
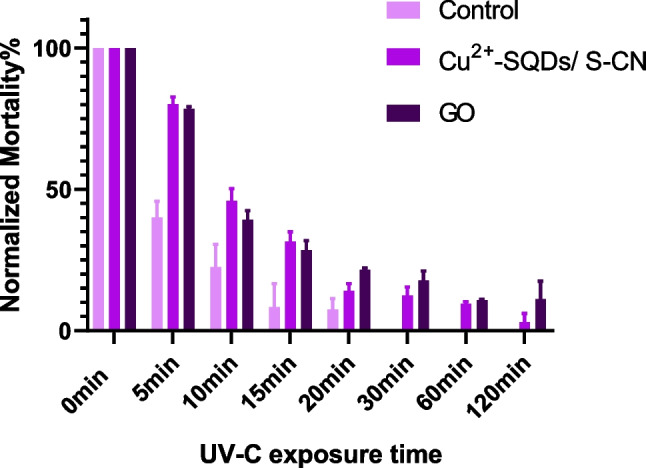
Larval mortality of Cry1Ab (control), GO emulsion, and Cu^2+^-SQDs/S-CN emulsion after UV radiation at different times (*n* = 3). The error bars were not drawn at time 0 because its mean was the value taken as a reference to correct mortalities

The samples obtained after the UV treatment time were tested for toxicity (Fig. 5). The number of dead larvae after the treatments was statistically analyzed (Table S1). After UVC exposure, for non-encapsulated Cry1Ab, the mortality value was reduced to 50% (half-life value) after just 4-min (FL_95_ 3.1–5.0) treatment, reduced to 90% at 10.4 min (FL_95_ 8.7–12), and became practically non-toxic (residual value, *R* = 0.005 ± 0.01% mortality) at the end of the experiment, indicating the high intensity of the treatment.

Both the emulsion stabilized with GO and the emulsion stabilized with Cu^2+^-SQDs/S-CN nanomaterials showed a slower reduction in mortality compared to the control: both encapsulations protected the toxicity of the Cry1Ab since the reduction of mortality rate (*K* parameter) was significantly lower (*P* < 0.002) than that of the controls. Indeed, the half-life values for GO and Cu^2+^-SQDs/S-CN emulsions were 7.1 min (FL_95_ 5.7 to 8.8) and 8.2 min (FL_95_ 6.8 to 9.8) respectively. These values indicated a 50% lower degradation rate for the encapsulated proteins. In fact, the reduction to 90% of initial mortality was achieved after 27 min (FL_95_ 22–41) and 27 min (FL_95_ 23–32) for each emulsion respectively, compared to the 10.4 min for the control. Moreover, the residual activity was also higher in both preparations than in the control (12- and fivefold for GO and Cu^2+^-SQDs/S-CN emulsions respectively). This protective effect could be related to the absorption wavelengths or much better temperature resistance. Summarizing, the results indicated that both emulsions stabilized by nanomaterials could protect Cry1Ab protoxin from UV radiation.

### Temperature protection

**Fig. 6:**
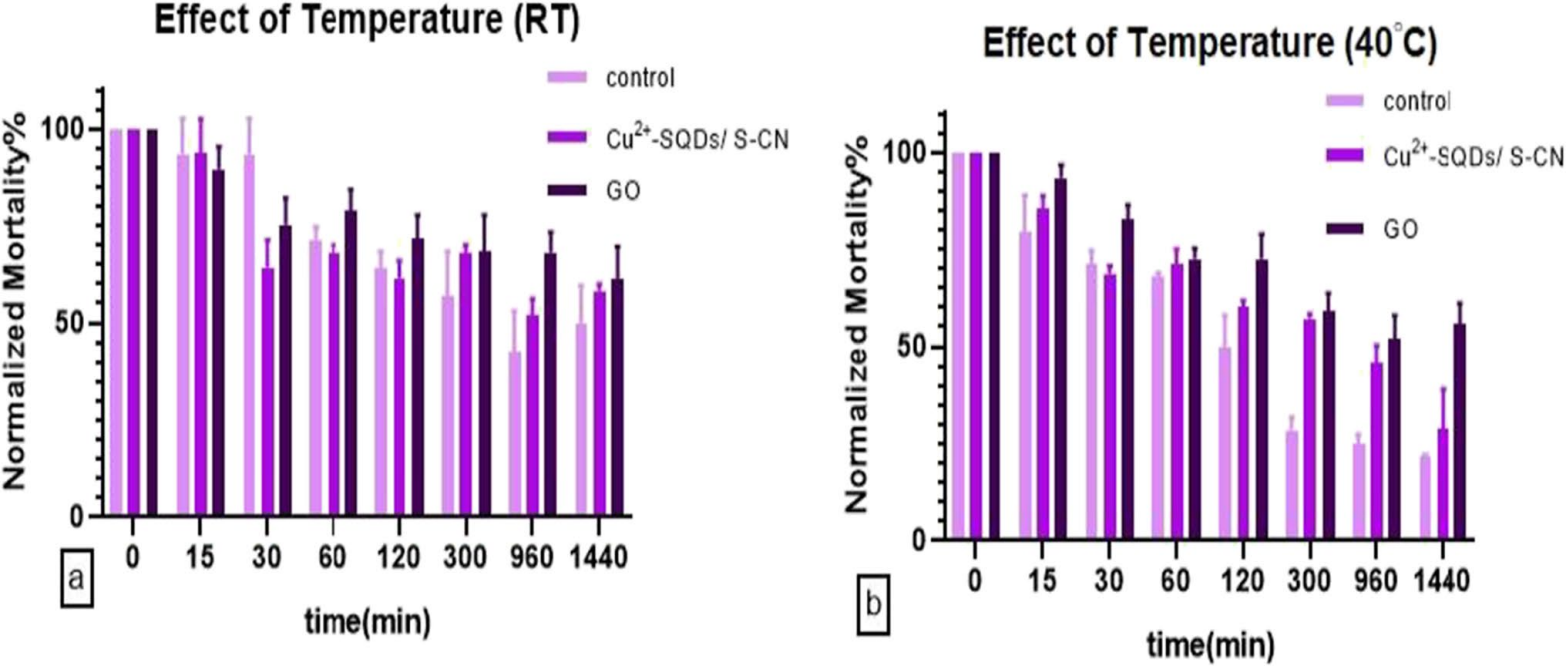
Larval mortality of Cry1Ab (control), GO emulsion, and Cu^2+^-SQDs/S-CN emulsion treated previously with room temperature (RT) (**a**) and 40°C (**b**) for different times (*n* = 3). The error bars were not drawn at time 0 because its mean was the value taken as a reference to correct mortalities

To determine the possible effect of nanomaterials in preventing the loss of insecticidal activity or degradation of Cry1Ab at room temperature (RT) and high temperatures (HT, 40 °C), the non-encapsulated and encapsulated Cry1Ab were treated at different times and tested for toxicity (Fig. 6).

Both encapsulations provided similar long-term toxicity protection at RT since showed significantly higher residual activity versus the control (*P* < 0.003) (Table S1). The effect of the HT treatment in terms of mortality in controls (Fig. 6b) was stronger than the RT treatment since reduced the initial mortality in the final experiment at about 50% (30% at RT one). Moreover, the long-term toxicity protection by encapsulation was reinforced since a significantly higher residual activity versus the control (*P* < 0.003) was obtained (Table S1). Differences in residual activity for both types of encapsulation were not statistically significant (*P* = 0.282).

## Discussion

Bt-based pesticidal products are not stable due to exposure to continuous sunlight and adverse temperature amongst other factors, which lead to reduced persistence and loss of toxicity (Leong et al. 1980). Several studies have been done to encapsulate, protect, and improve the resistance of Bt spores and their proteinaceous crystals (which contain the insecticidal Cry proteins) to environmental stresses (Aguilar-Meza et al. 2010; Khorramvatan et al. 2014; Naghavi et al. 2016; Barrera-Cortés et al. 2017; Xin et al. 2018; Eski et al. 2019).

In this study, the new encapsulated Cry1Ab with longer persistence was prepared using the Pickering emulsion technique stabilized by two different nanomaterials. Here, we describe pH-sensitive capsule-containing Cry1Ab prepared by the Pickering emulsion method. There are several advantages to these nanocapsules, including nanoparticles with light-absorbing properties, no need for organic solvents, faster Cry1Ab release in the alkaline environment (pH of the midgut of larvae) than in the acidic and neutral pH (Jalali et al. 2020), and maintenance of Cry1Ab bioactivity. Also, GO is not considered hazardous by the 2012 OSHA Hazard Communication Standard (29 CFR 1910.1200) (MSDS 2021). According to the Material Safety Data Sheet of ACS Material LLC, g-CN does not contain any components that are considered to be either persistent, bioaccumulative, and toxic, or very persistent and very bioaccumulative at a concentration of 0.1% or higher (Llc ACSM 2021). Additionally, studies show the safety of SQDs when administered up to a concentration of 2.4 mg/mL in vitro and 1.2 mg/mL in vivo (Priyadarshi et al. 2022). For Cu nanoparticles, the Safety Data Sheet according to Regulation 1907/2006 states: Acute toxicity LD50 oral—rat > 2500 mg/kg and LD50 dermal—rat > 2000 mg/kg (MSDS 2019). The amounts of nanomaterials used in all our experiments were below these limits. Using GO nanosheets and Cu^2+^-SQDs/ S-CN nanocomposite, we have attempted to develop a new nanoencapsulation method for protecting the insecticidal activity of solubilized Cry1Ab, more prone to be affected by environmental conditions than the Cry1Ab in the crystals.

The toxicity of the Cry1Ab protein-free or incorporated into the emulsions stabilized by GO nanosheets and Cu^2+^-SQDs/S-CN nanocomposite was tested, and the results showed that the toxicity of both Cry1Ab encapsulated materials was slightly higher. A synergistic effect of GO with chemical insecticides to control the lepidopteran pest *Spodoptera frugiperda* had been already observed (Li et al. 2022) and is probably due to a higher activation rate in the gut of the target insect as had been described for nanoencapsulated Cry11Aa (Pan et al. 2023).

The goal of this work was to increase the persistence of the solubilized Cry1Ab insecticidal protein from Bt in adverse conditions and to prolong the time during which it is toxic after exposure to UVC and 40°C temperature. Although 40 °C temperature does not exist in all regions, in all seasons, and the actual UV radiation in the field is not UVC (UVA and UVB are the UV radiations from sunlight), these conditions were chosen with the aim to apply drastic treatments which could accelerate the effects of radiation and temperature on Cry1Ab toxicity. SDS-PAGE results showed that the non-capsulated Cry1Ab was 90% degraded after just 15 min of treatment with UVC radiation and under the same conditions, both Cry1Ab encapsulated by nanomaterials seemed not to be affected. The bioassays performed after the UVC treatment showed that the toxicity was affected in both the free and the encapsulated Cry1Ab, but to a different extent, since the residual activity was up to 12-fold higher for the encapsulated ones. This protective effect against UV light is of the same order as the one described by Pan et al. (Pan et al. 2023) studying the bioactivity of pure Cry11Aa protected with nano-Mg(OH)_2_, against *Culex quinquefasciatus*. Also, Jalali et al. (Jalali et al. 2020) detected a bioactivity protective effect of about 1.9 times when they used GO nanosheets and Pickering emulsion of *Bt* subsp. *kurstaki* spores and crystals, against *Ephestia kuehniella* after UVA radiation treatment.

The effect of the temperature treatment on non-encapsulated and encapsulated Cry1Ab in terms of *O. nubilalis* mortality was weaker than the effect of UVC light since the Span values were lower (57% for UVC, 30% for RT, and 46% for HT, in mortality terms) and required longer times (4 min half-life for UVC and about 1.2 h for temperature treatments). In addition, it is remarkable that both types of particles used provide similar protection to temperature treatments (RT and HT), but for UVC treatment, GO nanosheets which had been described as an activator of trypsin-like serine proteases (Wang et al. 2021) were the most efficient nanomaterial in maintaining the toxicity of Cry1Ab and therefore in stabilizing the protein.

The UV and temperature are suggested as the most important factors causing Cry1Ab protein degradation in nature. In this work, it has been shown that the toxicity of the Cry1Ab protein protected by GO nanosheets and Cu^2+^-SQDs/S-CN nanocomposite persisted for more time than the non-protected Cry1Ab after RT (storage condition), UVC, or 40°C treatments. Thus, Cu^2+^-SQDs/S-CN nanocomposite, and especially GO nanosheets, could be promising protectants that provide good protection with longer survival against UV radiation and temperature. The use of these protectants is a promising option to apply in Bt treatments for field biological control of pests.

### Supplementary Information

Below is the link to the electronic supplementary material.

Supplementary file1 (PDF 277 KB)

## Data Availability

Available on reasonable request.
