# Supplementary material for: Enhancing insecticidal efficacy of Bacillus thuringiensis Cry1Ab through pH-sensitive encapsulation
Source: Appl Microbiol Biotechnol. 2023 Aug 26;107(20):6407–19. doi: 10.1007/s00253-023-12723-w (PMC10560195; doi:10.1007/s00253-023-12723-w)
Supplement: Supplementary file 1 — Supplementary file1 (PDF 277 KB) [file 253_2023_12723_MOESM1_ESM.pdf]

Enhancing insecticidal efficacy of *Bacillus thuringiensis* Cry1Ab through pH-sensitive encapsulation

Journal: Applied Microbiology and Biotechnology

Elham Jalali[1,2], Yolanda Bel[2], Shahab Maghsoudi[1], Ebrahim Noroozian[1], Baltasar Escriche[2,*]

[1] **Department of Chemistry, Shahid Bahonar University of Kerman, 76169-14111, Kerman, Iran. elham.jalali@yahoo.com, sahabmaghsoudi@uk.ac.ir, e_noroozian@uk.ac.ir**

[2] **Instituto BioTecMed, Department of Genetics, University of Valencia, 46100-Burjassot, Valencia, Spain. yolanda.bel@uv.es, baltasar.escriche@uv.es**

*Corresponding author

The supplementary figures correspond to optical microscopy images of Pickering emulsion stabilized by GO (Fig. S1) and Cu^{2+}-SQDs/S-CN (Fig. S2) with the oil/ aqueous.

The supplementary table (Table S1) reports the parameters of the dead larvae response to the different treatments adjusted to a first-order reaction and the statistical comparison among them.

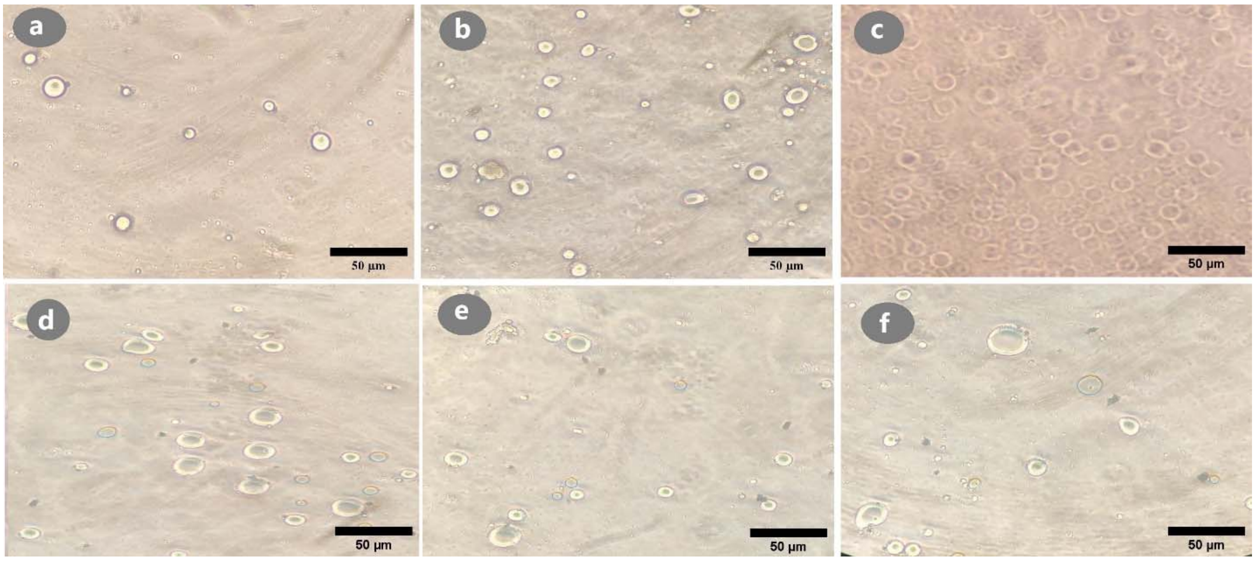

Fig. S1 Optical microscopy images of Pickering emulsion stabilized by GO with the oil/ aqueous volume ratio of a) 1:1, b) 2:1, C) 3:1, d) 4:1, e) 5:1, and f) 10:1. Scale bar represents 50 µm.

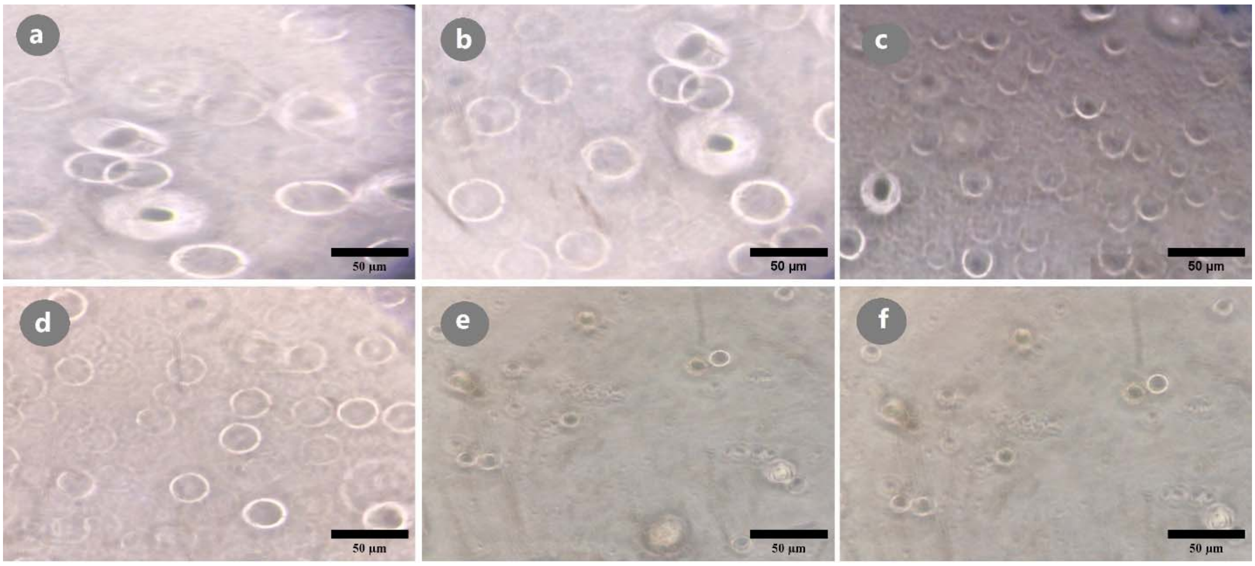

Fig. S2 Optical microscopy images of Pickering emulsion stabilized by Cu^{2+}-SQDs/S-CN with the oil/ aqueous volume ratio of a) 1:1, b) 2:1, C) 3:1, d) 4:1, e) 5:1, and f) 10:1 d) 4:1, e) 5:1, f) 10:1. Scale bar represents 50 µm.

Table S1 Statistical analyses of the degradation of the Cry1Ab protein non encapsulated (control) and encapsulated with GO emulsion (GO) and Cu^{2+}-SQDs/S-CN emulsion (SQDs/S-CN), after the treatments with ultraviolet light C (UVC), room temperature (RT), and 40 ºC (HT). Three replicates of each experiment were performed. The response of the treatment in terms of dead larvae data was analyzed as a first-order equation providing the parameters Span, R (both with dead larvae as a unit), and K (measured in min^{-1} for UVC treatment and h^{-1} for temperature treatments). Dead larvae numbers can be transformed in mortality by dividing them by the number of larvae used in each single assay (n=16). Parameter values are shown with ± SE (standard error). All analyses had 21 degrees of freedom and the r^2 ranged from 0.70-0.96. Parameters were compared under One-way ANOVA tests (2, 6 degrees of freedom) and compared in pairs with a Tukey's posttest. NA-Not applicable

	UVC			RT			HT		
	Control	GO	SQDs/S-CN	Control	GO	SQDs/S-CN	Control	GO	SQDs/S-CN
Span	9.19±0.42	8.73±0.44	11.81±0.49	4.86±0.66	3.30±0.49	4.41±0.62	6.90±0.52	4.16±0.39	5.87±0.71
K	0.17±0.02	0.10±0.01	0.08±0.01	0.59±0.22	1.93±0.67	2.11±0.43	0.51±0.11	0.65±0.16	0.47±0.16
R	0.08±0.21	0.95±0.26	0.41±0.31	4.52±0.45	6.27±0.24	6.15±0.23	1.93±0.37	5.31±0.26	4.77±0.51
One-way ANOVA statistics									
Parameters	Span	K	R	Span	K	R	Span	K	R
F-value	40.5	38.1	8.4	5.5	6.5	24.4	18.7	1.2	63.7
P-value	<0.001*	<0.001*	0.018*	0.044*	0.032*	0.001*	0.003*	0.3751	<0.001*
P-value Tukey's posttests									
Control vs GO	0.472	0.001*	0.016*	0.042*	0.062	0.002*	0.002*	NA	<0.001*
Control vs SQDs/S-CN	0.001*	0.001*	0.339	0.648	0.038*	0.003*	0.135	NA	<0.001*
GO vs SQDs/S-CN	<0.001*	0.488	0.099	0.132	0.918	0.896	0.022*	NA	0.282

*Data with significant differences in ANOVA (P-values < 0.05).
